# LncRNA LINC01605 Regulates Smooth Muscle Cell Functions and Participates in the Development of Aortic Dissection Through Regulating SGK1

**DOI:** 10.1111/jcmm.70963

**Published:** 2025-11-26

**Authors:** Mingliang Li, Ruonan Li, Zihe Zheng, Changbo Xiao, Quanlin Yang, Bo Chen, Xiaofu Dai

**Affiliations:** ^1^ Department of Cardiovascular Surgery Fujian Medical University Union Hospital Fuzhou China; ^2^ Key Laboratory of Cardio‐Thoracic Surgery (Fujian Medical University), Fujian Province University Fuzhou China; ^3^ Department of Cardiovascular Surgery The General Hospital of Ningxia Medical University Yinchuan China; ^4^ The Operating Room, Cancer Hospital, The General Hospital of Ningxia Medical University Yinchuan China; ^5^ Department of Cardiovascular Surgery Chest Hospital of Zhengzhou University Zhengzhou China; ^6^ Department of Cardiac Surgery Zhongshan Xiamen Hospital, Fudan University Xiamen China; ^7^ Department of Cardiovascular Surgery Gaozhou People's Hospital Gaozhou China

**Keywords:** aortic dissection, autophagy, LINC01605, phenotypic transformation, SGK1, vascular smooth muscle cells

## Abstract

Long noncoding RNAs (lncRNAs) are emerging as key regulators in cardiovascular diseases. This study investigated the role of lncRNA LINC01605 in aortic dissection (AD) pathogenesis through its effects on vascular smooth muscle cells (VSMCs). Bioinformatics analysis of GEO datasets (GSE107844, GSE147026) identified LINC01605 as differentially expressed in AD. Its expression was validated in human aortic tissues and VSMCs using RT‐qPCR and FISH. Functional assays (CCK‐8, Transwell, Western blot) assessed VSMC proliferation, migration, phenotypic switching and autophagy. SGK1 was predicted as a target via bioinformatics and confirmed by RIP assays. Ang II‐induced AD mice with LINC01605 knockdown were used for in vivo validation. LINC01605 was significantly upregulated in AD aortic tissues and VSMCs. Functional studies demonstrated that LINC01605 promoted VSMC proliferation, migration, invasion, phenotypic switching and autophagy, particularly under Ang II stimulation. Mechanistically, LINC01605 targeted SGK1 to regulate VSMC function. Knockdown of LINC01605 alleviated AD pathology in mice, modulating synthetic phenotype and autophagy markers. LINC01605 plays an important role in AD. It regulates the function of VSMCs by targeting SGK1 and promotes the pathological process of AD. LINC01605 may be a potential target for AD treatment, providing new directions for the mechanism research and treatment strategies of AD.

## 1 Introduction

Aortic dissection (AD), a life‐threatening cardiovascular disorder characterised by the separation of aortic wall layers, exhibits an estimated annual incidence of 3–6 cases per 100,000 individuals, with mortality rates exceeding 50% within 48 h if untreated [1, 2]. Histologically, AD progression is marked by degenerative changes in the medial layer, including extracellular matrix degradation and vascular smooth muscle cell (VSMC) depletion [3]. As the predominant cellular component of the aortic media, VSMCs play pivotal roles in maintaining vascular contractility, structural integrity and hemodynamic adaptation through phenotypic modulation [4]. Despite advances in surgical interventions, current pharmacological strategies remain limited by their inability to address the underlying molecular mechanisms driving VSMC dysfunction and medial degeneration, underscoring the urgent need for novel therapeutic targets [5, 6].

Emerging evidence highlights long noncoding RNAs (lncRNAs) as critical regulators of vascular pathophysiology. These > 200‐nucleotide transcripts modulate gene expression at epigenetic, transcriptional and posttranscriptional levels, influencing cellular processes ranging from proliferation to apoptosis [7, 8]. In AD pathogenesis, dysregulated lncRNAs have been implicated in endothelial dysfunction, inflammatory responses and VSMC phenotypic switching [9]. For instance, researchers observed elevated long noncoding RNA H19 expression in thoracic aorta tissues of AD patients and PDGF‐BB‐stimulated human aortic smooth muscle cells (HASMCs), which correlated with increased MMP‐2/9 levels, decreased contractile markers (α‐SMA/SM22α), and enhanced cell proliferation/migration [10]. Researchers demonstrated that long noncoding RNA SENCR overexpression suppressed vascular smooth muscle cell (VSMC) proliferation/migration and maintained contractile phenotype markers (e.g., myocardin) while downregulating synthetic phenotype genes, whereas SENCR knockdown exerted opposite effects. Mechanistically, SENCR functioned as a competitive endogenous RNA by directly binding to miR‐206, thereby relieving miR‐206‐mediated suppression of myocardin—a key transcriptional regulator of VSMC differentiation [11]. These findings position lncRNAs as potential diagnostic biomarkers and therapeutic candidates for AD management.

Autophagy a lysosome‐dependent degradation process, maintains VSMC homeostasis by eliminating damaged organelles and proteins [12]. Dysfunctional autophagy in VSMCs has been mechanistically linked to AD progression through dual mechanisms: excessive autophagy induces VSMC apoptosis and medial layer thinning, while impaired autophagy accelerates senescence and synthetic phenotype transition [13]. Recent studies demonstrate that pharmacological modulation of autophagy pathways (e.g., via mTOR inhibitors or AMPK activators) attenuates AD development in animal models, suggesting autophagy regulation as a promising therapeutic strategy [14]. However, the upstream regulators coordinating autophagy in AD‐associated VSMCs remain poorly characterised.

In this study, we integrated bioinformatics analysis of GEO datasets (GSE107844 and GSE147026) with functional validation to identify LINC01605 as a differentially expressed lncRNA in human AD tissues. Mechanistic investigations revealed that LINC01605 knockdown significantly inhibited VSMC migration, suppressed proliferation and restored autophagy. RNA pull‐down combined with RIP‐seq identified SGK1 as a direct binding partner mediating LINC01605's effects on VSMC biology. These findings establish the LINC01605/SGK1 axis as a novel regulatory circuit in AD pathogenesis, providing potential therapeutic targets for precision intervention.

## 2 Materials and Methods

### 2.1 Clinical Samples and Ethics

Human aortic tissues were collected from 30 aortic dissection (AD) patients and 30 healthy controls (HC) with informed consent. The study was approved by the Ethics Committee of the Fujian Medical University Union Hospital (Approval No. 2024KY178). Vascular smooth muscle cells (VSMCs) were isolated from tissues using collagenase digestion and identified by α‐SMA immunofluorescence staining [13].

### 2.2 Differentially Expressed lncRNA Screening

Differentially expressed lncRNAs in AD were screened from two GEO datasets (GSE107844 and GSE147026). The GSE107844 dataset included 3 normal control (NC) samples and 3 AD samples, while the GSE147026 dataset included 4 NC samples and 4 AD samples. Raw count data from these datasets were preprocessed and analysed using the DESeq2 package in R. Differentially expressed genes (DEGs) were identified with the following criteria: adjusted *p*‐value (*p*adj) < 0.05 and |log2FoldChange| ≥ 0.5 for volcano plot analysis. To specifically identify differentially expressed lncRNAs, a stricter threshold of *p*‐value (*p*adj) < 0.05 and |log2FoldChange| ≥ 1 was applied. Venn analysis was performed to identify common differentially expressed lncRNAs across the two datasets, and LINC01605 was identified as a significantly upregulated lncRNA in both datasets.

### 2.3 Target Gene Prediction and Validation

Target genes of LINC01605 were predicted using the ENCORI database (https://rnasysu.com/encori/). To validate the predicted targets, we intersected them with AD‐related differentially expressed mRNAs identified from the GSE107844 dataset and the Genecards database. This intersection analysis helped prioritise biologically relevant target genes for further investigation.

### 2.4 Data Preprocessing and Normalisation

Raw count data from the GSE107844 and GSE147026 datasets were preprocessed by filtering low‐expression genes (retaining genes with at least 10 counts in at least one group). The data were then normalised using the DESeq2 package, which estimates size factors to correct for sequencing depth differences and dispersion parameters for differential expression analysis. Multiple‐testing correction was performed using the Benjamini‐Hochberg procedure to control the false discovery rate (FDR) at a threshold of < 0.05.

### 2.5 Cell Culture and Lentiviral Transfection

Human aortic vascular smooth muscle cells (VSMCs, CP‐H116) were purchased from Procell Life Science & Technology Co. Ltd., Wuhan, China. The cells were cultured in Ham's F‐12 K medium (PM150910) supplemented with 0.05 mg/mL vitamin C, 0.01 mg/mL insulin, 0.01 mg/mL transferrin, 10 ng/mL sodium selenite, 0.03 mg/mL ECGs, 10% fetal bovine serum (FBS), 10 mM HEPES, 10 mM TES and 1% penicillin–streptomycin (P/S), all of which were obtained from Procell. HA‐VSMCs were used for experiments at passages 3–5. For genetic manipulation, lentiviral vectors targeting LINC01605 (overexpression: OE‐LINC01605; knockdown: sh‐LINC01605) and SGK1 (OE‐SGK1 and sh‐SGK1) were purchased from Genechem Inc. (Shanghai, China). Scrambled shRNA (sh‐NC) and empty vector (OE‐NC) served as negative controls.

VSMCs were seeded in six‐well plates (5 × 10^4^ cells/well) and transfected 24 h later with lentivirus at a multiplicity of infection (MOI) of 20 (1 × 10^8^ TU/mL, 2 μL/well). The blank control group received an equivalent volume of fresh culture medium. After 72 h of transfection, cells were treated with Angiotensin II (Ang II, Sigma, St. Louis, MO, USA) at a concentration of 1000 nM (dissolved in sterile PBS) for 24 h to induce pathological stress.

### 2.6 RT‐PCR

Total RNA was extracted using TRIzol reagent (Invitrogen, Carlsbad, CA, USA). First‐strand cDNA was synthesised from 1 μg total RNA using the PrimeScript RT Master Mix (Takara, Shiga, Japan) following the manufacturer's protocol. qRT‐PCR was performed on an ABI 7500 Real‐Time PCR System (Applied Biosystems, USA) with SYBR Green PCR Master Mix (Toyobo, Osaka, Japan). Each 20 μL reaction contained 10 μL SYBR Green mix, 1 μL cDNA template, 0.5 μM forward and reverse primers (Table S1) and 8.5 μL nuclease‐free water. Cycling conditions: 95°C for 10 min (initial denaturation), followed by 40 cycles of 95°C for 15 s and 60°C for 30 s. β‐actin was the housekeeping gene. Relative expression levels were calculated using the $2^{-∆∆C_{t}}$ method. All reactions were performed in triplicate (technical replicates) for each biological sample.

### 2.7 Fluorescence In Situ Hybridization (FISH)

After fixation with 4% formaldehyde, cellular membranes of VSMCs were permeabilised using 0.3% Triton X‐100, followed by blocking with 5% bovine serum albumin (BSA) to minimise non‐specific binding. FITC‐labelled LINC01605 probes and Cy3‐labelled SGK1 probes (RiboBio, Guangzhou, China) were hybridised to VSMCs overnight at 37°C. After washing with PBS, nuclei were counterstained with DAPI (Sigma, 1 μg/mL) for 5 min. Images were captured using a Zeiss LSM 880 confocal microscope (Carl Zeiss, Oberkochen, Germany) and analysed with ImageJ (NIH, MD, USA).

### 2.8 CCK‐8 Assay

VSMCs (5 × 10^3^ cells/well) were seeded in 96‐well plates. At 24, 48 and 72 h post transfection, 10 μL CCK‐8 reagent (Dojindo, Kumamoto, Japan) was added to each well. After 2 h of incubation, absorbance at 450 nm was measured using a BioTek Synergy H1 microplate reader (BioTek Instruments, VT, USA).

### 2.9 EdU Assay

Cells were incubated with 10 μM EdU (Beyotime, Shanghai, China) for 2 h, fixed with 4% PFA, and stained using the Click‐iT EdU Alexa Fluor 488 Kit (Invitrogen). Fluorescence images were acquired with an Olympus IX73 microscope (Olympus, Tokyo, Japan). Proliferation rates were quantified as the ratio of EdU‐positive cells to total DAPI‐stained nuclei.

### 2.10 Transwell Migration Assay

VSMCs (1 × 10^5^ cells) in serum‐free DMEM were added to the upper chamber of Transwell inserts (8 μm pores, Corning, NY, USA). The lower chamber contained DMEM with 10% FBS. After 24 h, migrated cells were fixed with methanol, stained with 0.1% crystal violet (Beyotime) and counted under a Nikon Eclipse Ti microscope (Nikon, Tokyo, Japan).

### 2.11 Scratch Wound Healing Assay

Confluent VSMC monolayers were scratched with a 200 μL sterile pipette tip. Wound closure was monitored at 0 and 24 h using a phase‐contrast microscope (Nikon). Migration distance was measured using ImageJ.

### 2.12 Animal Experiment

Male ApoE^−/−^ mice (8–10 weeks old, C57BL/6 background) were housed under standard SPF conditions and randomised into four groups (*n* = 8–10/group): Sham (ApoE^−/−^ mice infused with saline via subcutaneous osmotic minipumps [Alzet 2004] for 28 days), AD (mice receiving angiotensin II [Ang II, 1000 ng/kg/min] via osmotic pump to induce aortic dissection over 4 weeks), AD + sh‐NC (AD mice injected intravenously with scrambled shRNA lentivirus [1 × 10^8^ transduction units (TU), every 7 days]) and AD + sh‐LINC01605 (AD mice treated with LINC01605‐specific shRNA lentivirus [same dose/frequency]). Lentiviral delivery commenced 3 days prior to Ang II infusion to ensure target gene knockdown during AD progression, as adapted from published protocols [15]. Lentiviral particles (in 0.1 mL PBS) were administered via the tail vein 3 days before minipump implantation to ensure preemptive gene knockdown. Mice were anaesthetised with 2% isoflurane (Vet One), and osmotic pumps were subcutaneously implanted after a dorsal skin incision. Ang II/saline infusion continued for 28 days. At endpoint, mice were euthanised with pentobarbital sodium (100 mg/kg), and aortas were harvested. AD was confirmed by histopathology (H&E staining) showing medial rupture, thrombosis or elastic fibre fragmentation. All procedures complied with NIH guidelines and were approved by the Fujian Medical University Union Hospital Ethics Committee (Approval No. IACUC FJMU 2024–0277).

### 2.13 Western Blot

Proteins were extracted with RIPA lysis buffer (Beyotime) containing protease inhibitors. Lysates (30 μg/lane) were separated on 10% SDS‐PAGE gels and transferred to PVDF membranes (Millipore, MA, USA). Membranes were blocked with 5% nonfat milk and probed overnight at 4°C with primary antibodies [α‐SMA (14395‐1‐AP, 1:6000, Proteintech), SM22α (ab14106, 1:1000, Abcam), MMP‐2 (10373‐2‐AP, 1:600, Proteintech), MMP‐9 (ab137867, 1:1000, Abcam), p62 (ab155686, 1:500, Abcam), LC3B (ab222776, 1:200, Abcam), SGK1 (28454‐1‐AP, 1:600, Proteintech), GAPDH (ab181603, 1:10,000, Abcam)]. HRP‐conjugated secondary antibodies (SA00001‐2, 1:5000, Proteintech) and ECL substrate (Millipore) were used for detection. Band intensities were quantified with ImageLab (Bio‐Rad, CA, USA).

### 2.14 Tandem Fluorescent‐Tagged LC3 Reporter Assay

Cells were transfected with a plasmid encoding tandem fluorescent‐tagged LC3 (GFP‐mRFP‐LC3) using Lipofectamine 2000 according to the manufacturer's instructions. After 24 h, cells were treated with sh‐LINC01605, Ang II or CQ as described above. Confocal microscopy was used to visualise the localization of GFP‐mRFP‐LC3 puncta. Images were captured using a confocal microscope (model name), and the fluorescence intensity was quantified using ImageJ software. The number of GFP − mRFP+ and GFP + mRFP+ puncta per cell was counted and averaged across multiple fields of view.

### 2.15 Statistical Analysis

All experiments were performed in triplicate (*n* = 3). The experimental outcomes were processed and analysed utilising SPSS 13 (Statistical Package for the Social Sciences). The data derived from measurements are presented as the mean ± standard deviation (SD). To evaluate the differences in means between two distinct groups, an independent samples *t*‐test was employed. For comparisons involving more than two groups, a one‐way analysis of variance (ANOVA) was conducted. Statistical significance was established at a threshold of *p* < 0.05.

## 3 Results

### 3.1 LINC01605 Is Highly Expressed in the Aortic Wall Tissue of Patients With Thoracic Aortic Dissection (TAD)

**FIGURE 1:**
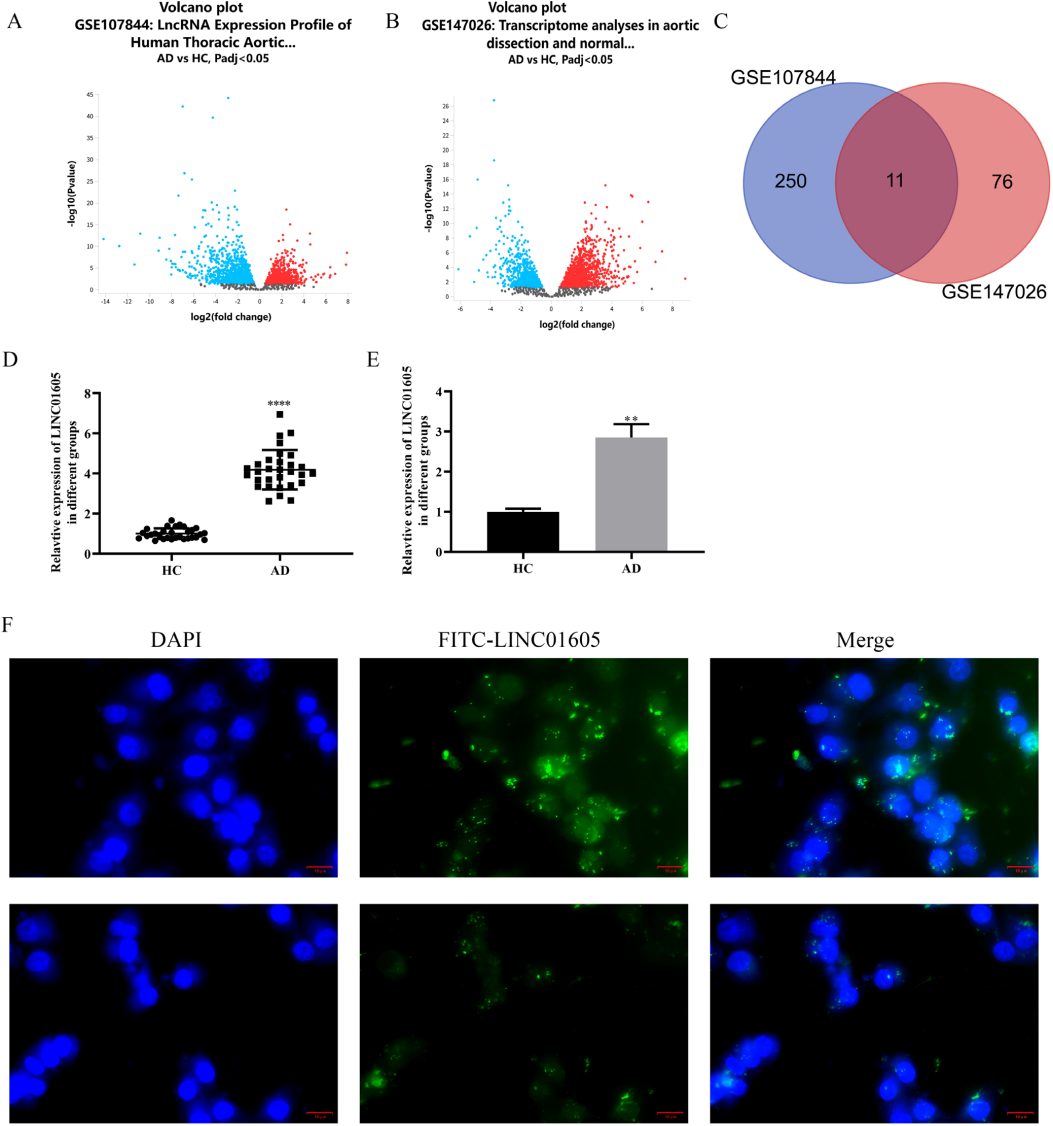
Identification and validation of LINC01605 as a highly expressed lncRNA in aortic dissection (AD). (A, B) Volcano plots of differentially expressed lncRNAs in AD aortic tissues vs. normal controls from GEO datasets GSE107844 (937 DE‐lncRNAs: 435 upregulated, 502 downregulated) and GSE147026 (87 DE‐lncRNAs: 35 upregulated, 52 downregulated). Red/blue dots indicate upregulated/downregulated lncRNAs (|log2FC| > 0.5, *p* < 0.05). (C) Venn diagram showing 13 overlapping lncRNAs from GSE107844 and GSE147026. (D) RT‐PCR validation of LINC01605 expression in aortic tissues from AD patients (*n* = 30) vs. normal donors (*n* = 30). (E) LINC01605 expression in VSMCs isolated from AD patients vs. normal aortic donors. (F) Fluorescence in situ hybridization (FISH) showing cytoplasmic localization of LINC01605 (green) in VSMCs. Nuclei counterstained with DAPI (blue). Scale bar: 20 μm. ****p < 0.0001 vs. HC, **p < 0.01 vs. HC. AD, aortic dissection; FC, fold change; FISH, fluorescence in situ hybridization; lncRNA, long noncoding RNA; RT‐PCR, reverse transcription polymerase chain reaction; VSMCs, vascular smooth muscle cells.

We obtained the datasets GSE107844 and GSE147026 from the GEO database and analysed the differentially expressed long noncoding RNAs (lncRNAs) in the aortic wall tissue of TAD patients and the normal aortic wall tissue of healthy donors from these datasets. GSE107844 identified a total of 2164 differentially expressed genes (DEGs), including 878 upregulated and 1286 downregulated genes (Figure 1A). GSE147026 identified a total of 2738 DEGs, including 1545 upregulated and 1193 downregulated genes (Figure 1B). A total of 261 differentially expressed lncRNAs were identified in GSE107844, including 105 upregulated and 156 downregulated ones. In GSE147026, 87 differentially expressed lncRNAs were obtained, with 35 upregulated and 52 downregulated. By taking the intersection of the differentially expressed lncRNAs in GSE107844 and GSE147026, 11 overlapping genes were identified through Venn analysis (Figure 1C). Among these genes, LINC01605 caught our attention. Through RT‐PCR detection, we found that it was abnormally highly expressed in the aortic wall tissue of patients with aortic dissection compared with normal aortic wall tissue (Figure 1D). Further, we isolated vascular smooth muscle cells (VSMCs) from the aortic wall tissue of patients with aortic dissection and normal aortic donors. Similarly, LINC01605 was overexpressed in VSMCs derived from patients with aortic dissection (Figure 1E). Moreover, through fluorescence in situ hybridization (FISH) localization, LINC01605 was found in the cytoplasm and nucleus (Figure 1F).

### 3.2 LINC01605 Promotes Cell Proliferation, Migration, Invasion, Phenotypic Transformation and Autophagy in Ang II‐Induced and Non‐Induced VSMCs

**FIGURE 2:**
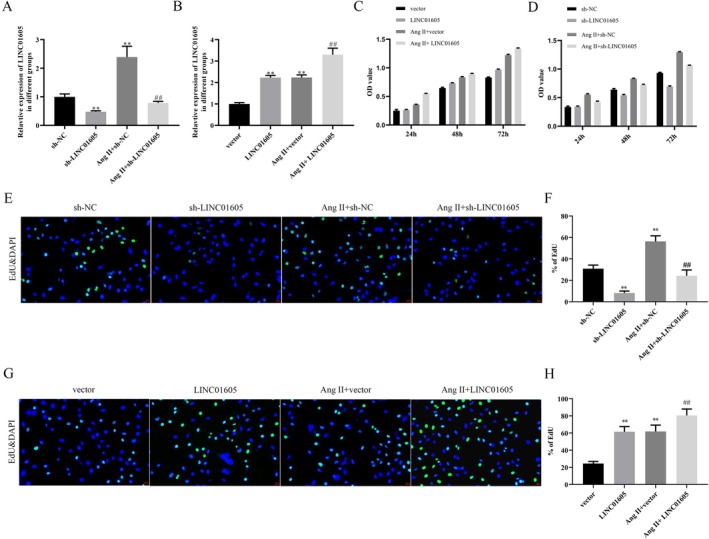
LINC01605 promotes proliferation in VSMCs under Ang II‐induced and basal conditions. (A, B) Expression of LINC01605 in VSMCs after Ang II treatment (24 h, 1 μM) or transfection with LINC01605 shRNA/overexpression plasmid. RT‐qPCR data normalised to GAPDH. (C–H) Proliferation of VSMCs assessed by CCK‐8 assay and EdU. Ang II (1 μM) enhanced proliferation, which was reversed by LINC01605 knockdown and further amplified by LINC01605 overexpression. Similar trends were observed under basal conditions (no Ang II). **p < 0.01 vs. sh‐NC or vector, ^##^ *p* < 0.01 vs. Ang II + sh‐NC or Ang II + vector.

**FIGURE 3:**
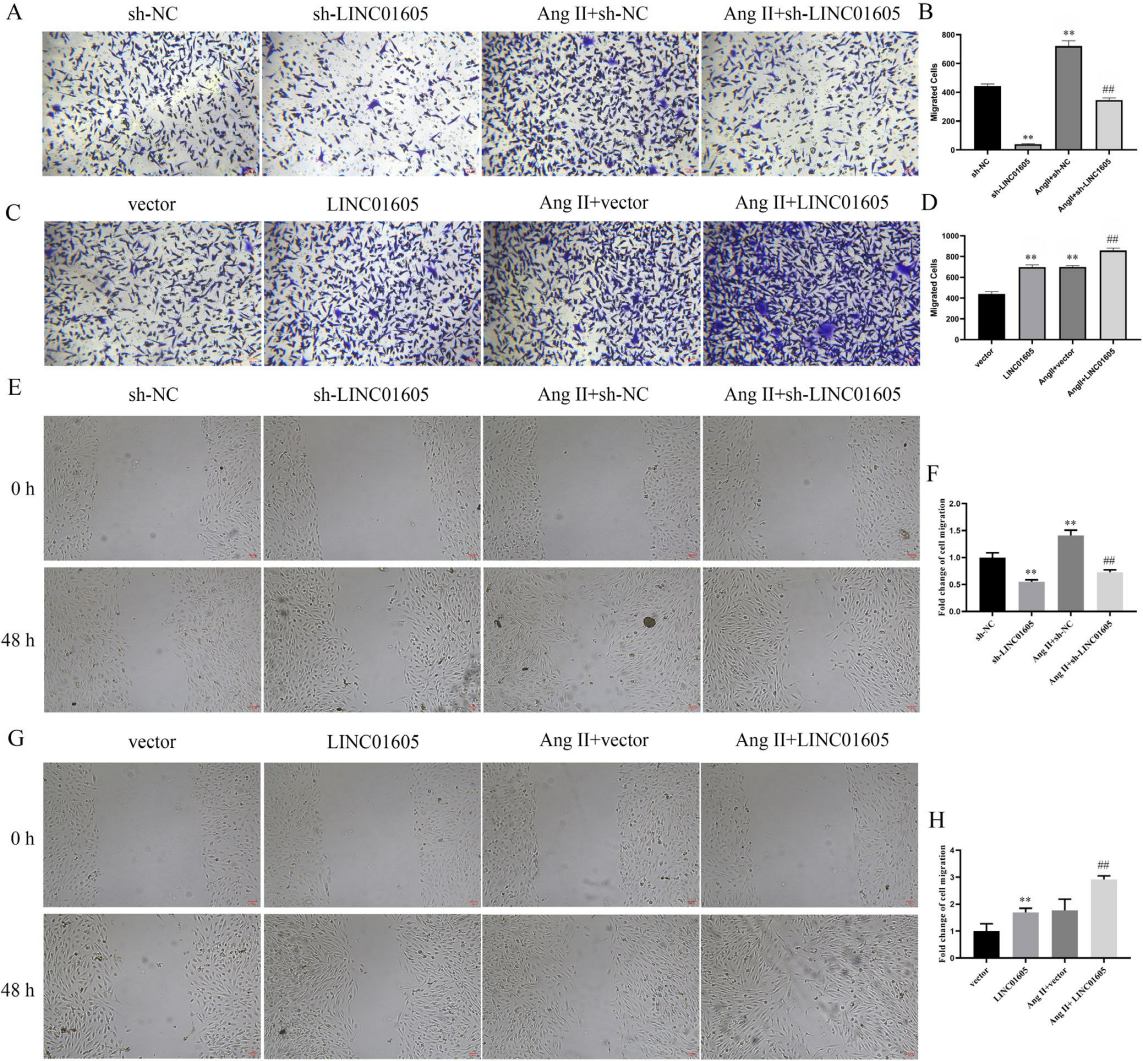
LINC01605 promotes migration in VSMCs under Ang II‐induced and basal conditions. (A, D) The cell migration was detected by transwell migration assays. Ang II stimulation increased VSMC migration, which was exacerbated by LINC01605 overexpression and suppressed by knockdown. LINC01605 alone (without Ang II) also promoted migration. Representative images (left) and quantification (right). (E, H) The cell migration was detected by scratch assays. **p < 0.01 vs. sh‐NC or vector, ^##^ *p* < 0.01 vs. Ang II + sh‐NC or Ang II + vector.

**FIGURE 4:**
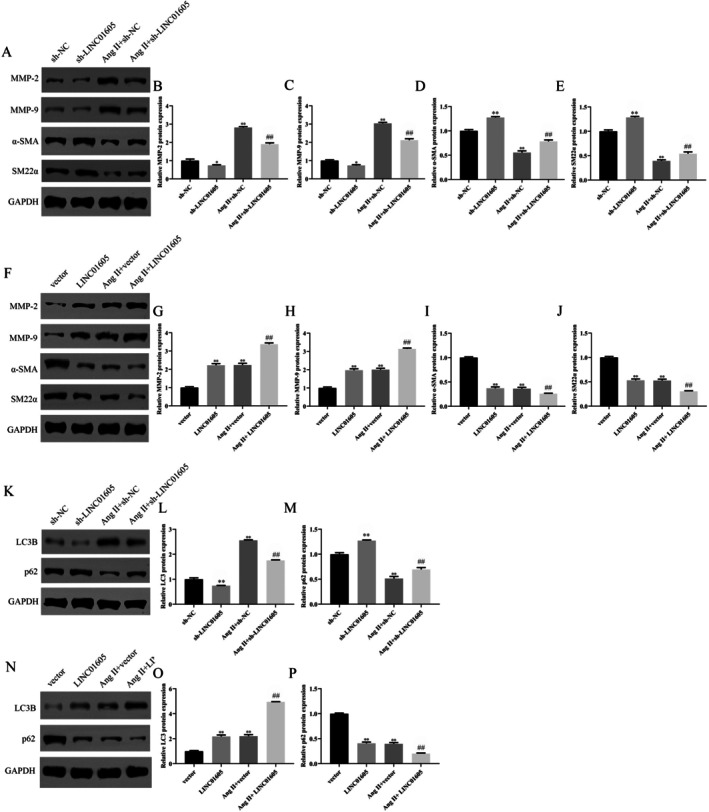
LINC01605 promotes phenotypic transformation and autophagy in VSMCs under Ang II–induced and basal conditions. (A–P) Western blot analysis of matrix metalloproteinases (MMP‐2, MMP‐9), phenotypic markers (α‐SMA, SM22α) and autophagy‐related proteins (LC3B, p62). LINC01605 overexpression downregulated contractile markers (α‐SMA, SM22α) and p62, while upregulating LC3B and MMPs. Knockdown reversed these effects. Quantification normalised to GAPDH. Data shown as mean ± SD. *p < 0.05 vs. sh‐NC or vector, **p < 0.01 vs. sh‐NC or vector, ^##^ *p* < 0.01 vs. Ang II + sh‐NC or Ang II + vector. Ang II, angiotensin II; CCK‐8, Cell Counting Kit‐8; LC3B, microtubule‐associated protein 1A/1B‐light chain 3B; RT‐qPCR, reverse transcription quantitative polymerase chain reaction; VSMCs, vascular smooth muscle cells; α‐SMA, alpha‐smooth muscle actin.

We explored the role of LINC01605 in VSMCs by knocking down and overexpressing LINC01605. Ang II significantly upregulated the expression of LINC01605 in VSMCs. After transfection, the expression of LINC01605 was significantly downregulated and upregulated, providing a reliable model for subsequent functional studies (Figure 2A,B). In terms of cell proliferation, Ang II treatment significantly enhanced the proliferation ability of VSMCs. Low expression of LINC01605 reversed this effect, while overexpression further enhanced it. In the absence of Ang II stimulation, the effect of LINC01605 on VSMC proliferation was similar to that under Ang II stimulation (Figure 2C–H). In terms of cell migration, Ang II treatment significantly improved the migration ability of VSMCs, and overexpression of LINC01605 further enhanced these effects. Similarly, in the absence of Ang II, overexpression of LINC01605 also significantly promoted the migration of VSMCs (Figure 3A–H). However, the effect of LINC01605 knockdown was opposite to that of LINC01605 overexpression (Figure 3A–H). The results of western blot showed that the expression of LINC01605 was positively correlated with the expression of MMP‐2, MMP‐9 and LC3B, and negatively correlated with the expression of α‐SMA, SM22α and p62 (Figure 4A–P). These results indicate that LINC01605 promotes the phenotypic transformation and autophagy of VSMCs with or without Ang II induction. The results of immunofluorescence further confirmed that LINC01605 promotes the occurrence of autophagy. The autophagic flux assay was employed to further validate that LINC01605 inhibits autophagy. In the Western Blot (WB) experiments, after the addition of chloroquine (CQ), both the sh‐LINC01605 group and the Ang II + sh‐LINC01605 group exhibited a significant increase in LC3B and p62 levels (Figure S1A–C). This indicates the accumulation of autophagosomes and the blockade of autophagic flux. The colocalization results of LC3‐mRFP and LC3‐GFP demonstrated that in the Sh‐LINC01605 group, there were fewer mRFP single‐fluorescence spots (red) and more GFP‐mRFP colocalization spots (yellow). This suggests an impairment in the fusion of autophagosomes with lysosomes (Figure S1D–G). After the addition of CQ, the number of mRFP single‐fluorescence spots (red) slightly increased, while the number of GFP‐mRFP colocalization spots (yellow) further increased. In the Ang II + sh‐LINC01605 group, the number of mRFP single‐fluorescence spots (red) increased, whereas the number of GFP‐mRFP colocalization spots (yellow) decreased, indicating enhanced fusion of autophagosomes with lysosomes. Following the addition of CQ, the number of mRFP single‐fluorescence spots (red) significantly increased, as did the number of GFP‐mRFP colocalization spots (yellow) (Figure S1D–G). These findings suggest that LINC01605 may regulate autophagy levels by inhibiting autophagic flux.

### 3.3 LINC01605 Promotes the Migration, Growth and Autophagy of VSMCs by Targeting SGK1

**FIGURE 5:**
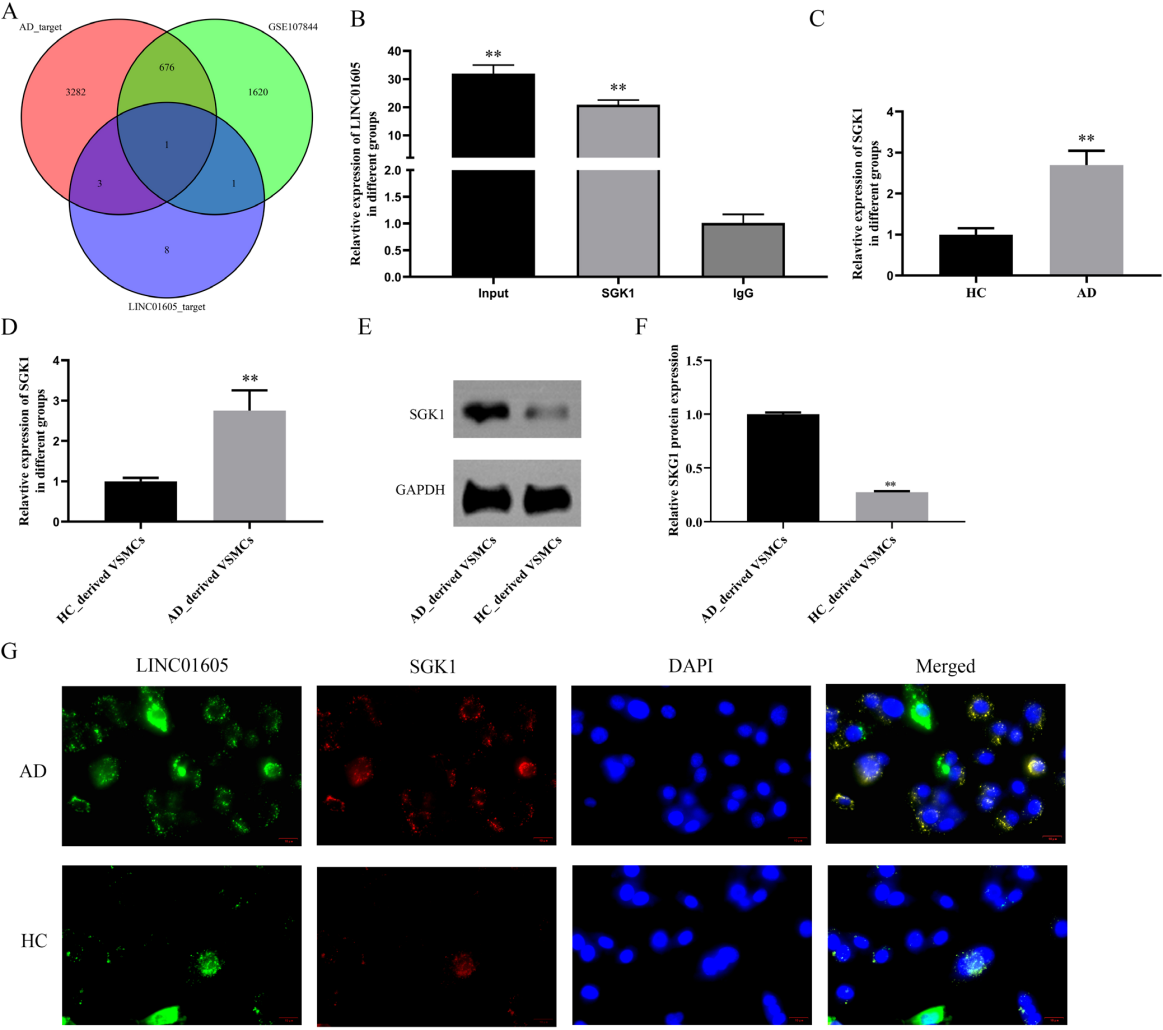
LINC01605 targets SGK1. (A) Venn diagram illustrating the intersection of LINC01605‐predicted targets, AD‐predicted targets and differentially expressed mRNAs in AD from the GSE107844 dataset, identifying SGK1 as a candidate target. (B) RNA immunoprecipitation (RIP) assay showing significant enrichment of LINC01605 by SGK1. (C–F) RT‐qPCR and Western blot analysis demonstrating SGK1 overexpression in aortic tissues and VSMCs from AD patients vs. normal donors (**p < 0.01). (G) Fluorescence in situ hybridization (FISH) revealing cytoplasmic colocalization of LINC01605 (FITC/green) and SGK1 (Cy3/red) in VSMCs. Nuclei counterstained with DAPI (blue). Scale bar: 20 μm. **p < 0.01 vs. IgG, **p < 0.01 vs. HC, **p < 0.01 vs. HC_derived VSMCs. AD, aortic dissection; ChIP, chromatin immunoprecipitation; VSMCs, vascular smooth muscle cells.

**FIGURE 6:**
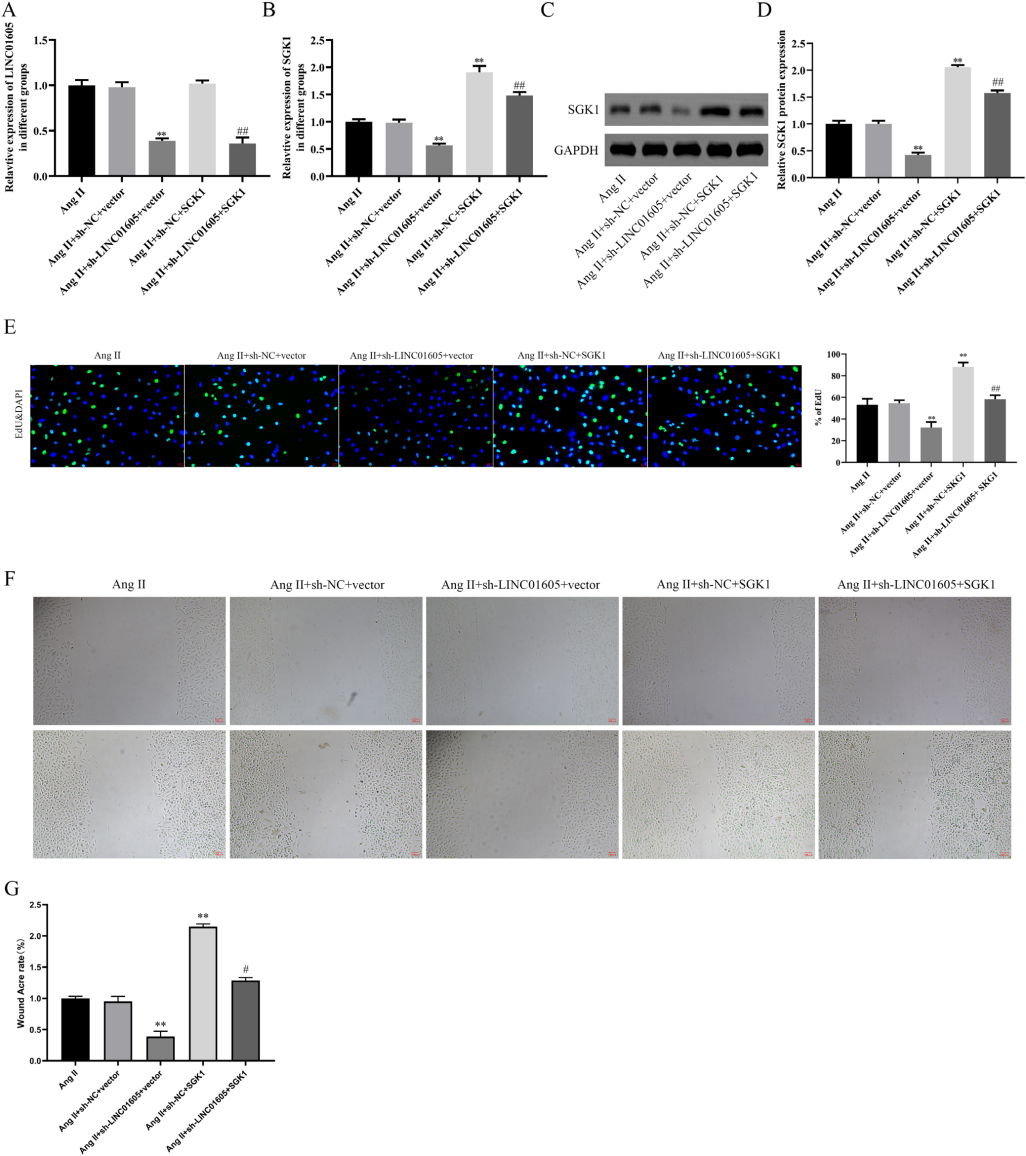
LINC01605 regulates VSMC proliferation and migration by targeting SGK1. (A–D) Effect of LINC01605 knockdown and SGK1 overexpression on SGK1 expression. Sh‐LINC01605 reduced SGK1 mRNA and protein levels, rescued by SGK1 plasmid transfection. (E) EdU assay showing LINC01605 knockdown suppressed VSMC proliferation, partially reversed by SGK1 overexpression. (F, G) Scratch assays. LINC01605 knockdown inhibited VSMC migration/invasion, rescued by SGK1 overexpression. **p < 0.01 vs. Ang II; ##*p* < 0.01 vs. Ang II + LINC01605 + vector.

**FIGURE 7:**
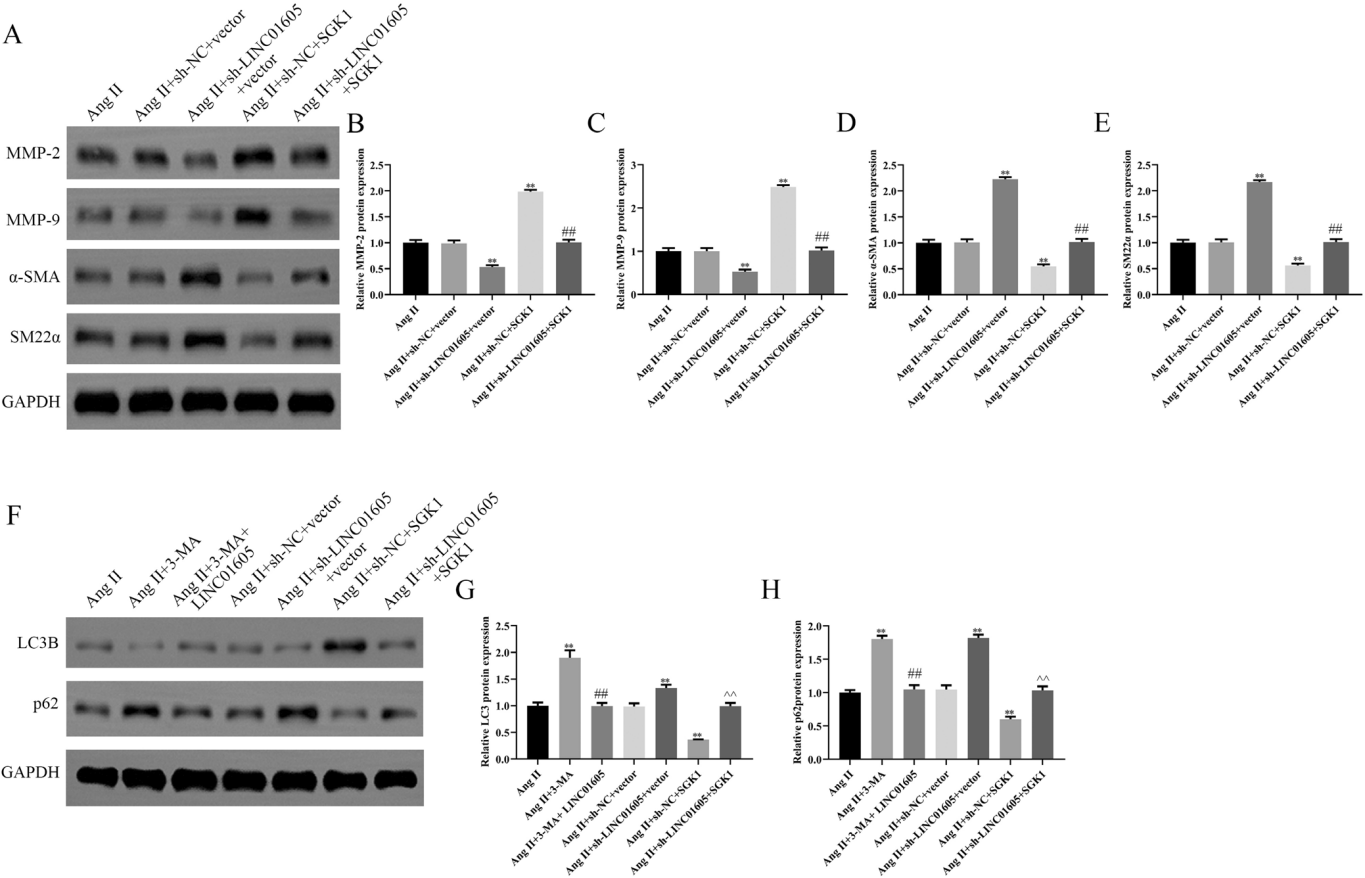
LINC01605 Regulates VSMC phenotypic transformation and autophagy by targeting SGK1. (A–H) Western blot analysis of phenotypic markers (α‐SMA, SM22α), matrix metalloproteinases (MMP‐2, MMP‐9) and autophagy‐related proteins (LC3B, p62). LINC01605 knockdown reduced MMPs and LC3B while increasing α‐SMA, SM22α and p62; SGK1 overexpression reversed these effects. Quantification normalised to GAPDH. **p < 0.01 vs. Ang II; ^##^ *p* < 0.01 vs. Ang II + LINC01605 + vector. α‐SMA, alpha‐smooth muscle actin; LC3B, microtubule‐associated protein 1A/1B‐light chain 3‐phosphatidylethanolamine conjugate.

In this study, we predicted potential targets of LINC01605 using the ENCORI database, obtained Alzheimer's disease (AD)‐related genes from Genecards, and identified differentially expressed genes from the GSE107844 dataset. The intersection of genes derived from these three approaches was determined through Venn analysis, leading to the identification of SGK1 as a candidate target (Figure 5A). We hypothesised that LINC01605 might affect the migration, growth and autophagy of VSMCs by targeting the key regulatory factor SGK1. To verify this hypothesis, a RIP assay was performed, and the results showed that LINC01605 could be significantly enriched by SGK1 (Figure 5B). Moreover, SGK1 was overexpressed in the aortic wall tissues and VSMCs of patients with aortic dissection (Figure 5C). SGK1 was overexpressed in VSMCs derived from patients with aortic dissection (Figure 5D–F). FISH colocalization analysis revealed that LINC01605 and SGK1 were coexpressed in the cytoplasm (Figure 5G). We have already conducted functional experiments to validate the role of SGK1 in AD in vitro. These data are provided in the Supporting Information (e.g., Figures S2–, S4). Specifically, our results demonstrate that knockdown or overexpression of SGK1 significantly affects cellular processes relevant to TAD pathogenesis, such as vascular smooth muscle cell (VSMC) migration, proliferation and autophagy. Knockdown of LINC01605 significantly reduced the mRNA and protein levels of SGK1, and overexpression of SGK1 could alleviate this effect (Figure 6A–D). In terms of cell growth, the CCK‐8 assay showed that low expression of LINC01605 significantly inhibited the proliferation of VSMCs, while overexpression of SGK1 attenuated this inhibitory effect (Figure 6E). In terms of cell migration, the scratch assay indicated that knockdown of LINC01605 significantly inhibited the migration ability of VSMCs, while overexpression of SGK1 reversed this effect (Figure 6F,G). In terms of VSMC phenotypic transformation and autophagy regulation, by detecting the expression of phenotype‐related proteins (α‐SMA, SM22α, MMP‐2 and MMP‐9) and autophagy‐related proteins (such as LC3B, p62), we found that inhibition of LINC01605 expression significantly attenuated the phenotypic transformation and autophagic activity of VSMCs, while overexpression of SGK1 inhibited this effect (Figure 7A–H).

### 3.4 Knockout of LINC01605 Alleviates the Progression of AD In Vivo

**FIGURE 8:**
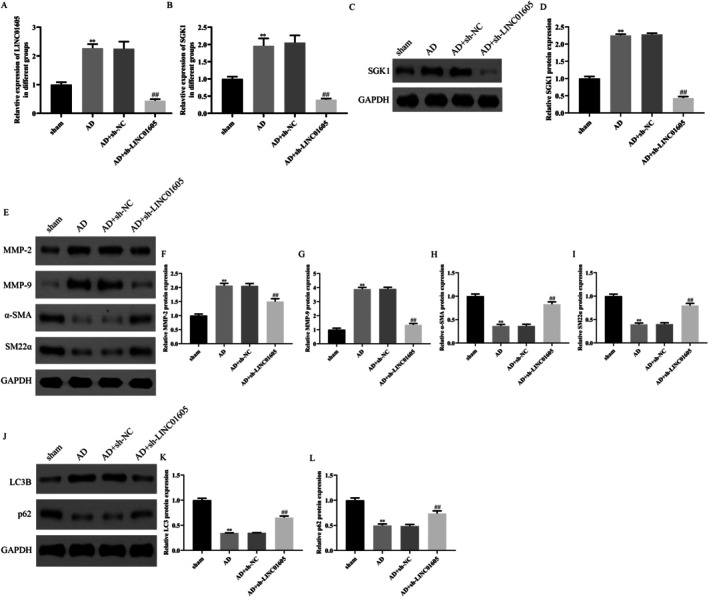
Knockdown of LINC01605 alleviates AD progression in vivo. (A–D) RT‐qPCR and Western blot analysis showing overexpression of LINC01605 and SGK1 in aortic tissues of AD mouse models compared to controls. LINC01605 knockdown via siRNA‐lentivirus significantly reduced their expression (**p < 0.01 vs. control; ^##^ *p* < 0.01 vs. AD model). (E–L) Western blot analysis of synthetic phenotype markers (MMP‐2, MMP‐9), contractile markers (α‐SMA, SM22α) and autophagy‐related proteins (LC3B, p62) in aortic tissues. Ang II treatment upregulated MMPs and LC3B while downregulating α‐SMA, SM22α and p62. LINC01605 knockdown reversed these changes (**p < 0.01 vs. sham; ^##^ *p* < 0.01 vs. AD + sh‐NC). AD, aortic dissection; Ang II, angiotensin II; ApoE^−/−^, apolipoprotein E‐deficient; RT‐qPCR, reverse transcription quantitative polymerase chain reaction; α‐SMA, alpha‐smooth muscle actin; LC3B, microtubule‐associated protein 1A/1B‐light chain 3B.

EVG and HE staining were utilised to detect pathological changes. The results of EVG staining demonstrated that the sham group exhibited a normal vascular wall structure with well‐organised elastic fibres. In contrast, the AD group showed evident disruption and disarray of elastic fibres. The AD + sh‐NC group did not exhibit significant improvement in the degree of lesion. However, the AD + sh‐LINC01605 group displayed a more orderly elastic fibre arrangement compared to both the AD and AD + sh‐NC groups, suggesting that the knockdown of LINC01605 may contribute to the restoration of the elastic fibre structure in the vascular wall (Figure S5A). The findings of HE staining indicated that the sham group had a normal vascular wall structure without any infiltration of inflammatory cells. The AD group, however, presented with substantial infiltration of inflammatory cells and proliferation of smooth muscle cells. There was no noticeable improvement in the inflammatory response in the AD + sh‐NC group. Conversely, the AD + sh‐LINC01605 group exhibited reduced inflammatory cell infiltration and decreased vascular wall thickness, indicating that the knockdown of LINC01605 aids in mitigating the pathological changes induced by AD (Figure S5B). We found that LINC01605 and SGK1 were overexpressed in the AD mouse model, and the transfection of lentivirus carrying small interfering RNA targeting LINC01605 significantly reduced their expression (Figure 8A–D). Next, we further evaluated the expression of synthetic phenotype‐related and autophagy‐related markers in the mouse aortas. In the model group, the expression of MMP‐2, MMP‐9 and LC3B was significantly increased, while the expression of α‐SMA, SM22α and p62 was significantly decreased. Low expression of sh‐LINC01605 could eliminate this effect (Figure 8E–L).

## 4 Discussion

AD represents a life‐threatening cardiovascular condition with limited therapeutic options, underscoring the urgent need to elucidate its underlying molecular mechanisms and identify novel therapeutic targets. In this study, we identified LINC01605, a previously uncharacterised long noncoding RNA (lncRNA), as a critical regulator of vascular smooth muscle cell (VSMC) dysfunction and aortic remodelling in AAD pathogenesis. We found that LINC01605 was significantly upregulated in AAD patient tissues and was functionally associated with VSMC proliferation, migration, invasion, phenotypic switching and autophagy. In vitro experiments demonstrated that LINC01605 knockdown attenuated Ang II‐induced VSMC dysfunction, while its overexpression exacerbated these effects. Furthermore, in vivo silencing of LINC01605 via lentiviral delivery markedly alleviated Ang II‐induced aortic injury in ApoE^−/−^ mice, as evidenced by reduced autophagy activation, preserved contractile phenotype markers (α‐SMA/SM22α) and suppressed MMP‐2/9 activity. Mechanistically, LINC01605 exerts its pathological effects by targeting SGK1, a key regulator of autophagy and VSMC homeostasis. These findings align with emerging evidence that lncRNAs, such as lnc‐OIP5‐AS1 and GAS5, modulate AAD progression through diverse molecular pathways [16, 17]. Our study thus highlights the LINC01605‐SGK1 axis as a novel regulatory node in AD pathogenesis and underscores the therapeutic potential of targeting lncRNA networks for AAD treatment.

Long noncoding RNAs (lncRNAs), defined as non‐protein‐coding transcripts exceeding 200 nucleotides, have emerged as critical regulators of gene expression through diverse mechanisms, including chromatin remodelling and posttranscriptional modulation [18, 19]. Recent studies have begun to unravel their roles in aortic pathologies. For instance, Cai et al. identified long noncoding RNA SENCR overexpression inhibited Ang‐II‐induced VSMC apoptosis, reduced matrix metalloproteinase (MMP)‐2/9 expression, and increased tissue inhibitor of metalloproteinases 1 (TIMP‐1) levels, thereby attenuating extracellular matrix degradation. In a mice model, SENCR upregulation ameliorated aortic wall pathological changes, preserved elastic fibre integrity and suppressed VSMC apoptosis [20]. These collective gaps underscore the pressing need to systematically delineate how specific lncRNAs mechanistically govern vascular smooth muscle cell (VSMC) dysfunction—a central driver of aortic remodelling. Addressing this, our study leveraged GEO database mining to identify LINC01605 as a novel lncRNA markedly upregulated in AAD tissues. LINC01605 expression correlated with adverse vascular phenotypes, including enhanced VSMC proliferation, migration and autophagy dysregulation. Functionally, in vitro knockdown of LINC01605 reversed Ang II‐induced VSMC synthetic phenotype switching and matrix degradation, while in vivo silencing attenuated aortic dissection severity in preclinical models. Our findings not only resolve prior ambiguities regarding lncRNA‐mediated VSMC regulation but also position LINC01605 as a pivotal orchestrator of AD pathogenesis, bridging the gap between transcriptional dysregulation and clinical vascular pathology.

The pathogenesis of aortic dissection involves multiple pathological processes of abnormal function of vascular smooth muscle cells (VSMCs) and imbalance of extracellular matrix (ECM) homeostasis [21]. Studies have shown that autophagy deficiency in VSMCs leads to the accumulation of misfolded proteins and mitochondrial dysfunction through the activation of the mTORC1 signalling pathway [22]. This metabolic disorder significantly increases the mechanical stress sensitivity of the vascular wall. Our findings indicate that Ang II can induce autophagy in VSMCs, and the expression of LINC01605 can further promote the occurrence of autophagy. In terms of proliferation regulation, Li et al. (2013) found that the abnormal activation of the Jagged‐1/Notch3 axis can induce excessive proliferation of VSMCs, forming a typical intimal false lumen structure [23]. Similarly, Ang II can enhance the proliferation of VSMCs, while the inhibition of LINC01605 can alleviate this effect. Notably, the phenotypic transformation of VSMCs plays an important role in the disease progression [24]. The transformation from the contractile type to the synthetic type mediated by the TGF‐β/Smad pathway, accompanied by the overexpression of MMP—2/9, leads to the rupture of elastic fibres, weakens the strength of the aortic wall, and ultimately makes the aorta prone to rupture and promotes the progression of AD [25]. In our study, Ang II can induce the phenotypic transformation of VSMCs, the overexpression of MMP—2/9, and cell migration, and LINC1605 has a positive effect on these impacts.

LncRNAs play a dual role in regulating mRNA expression. They can either promote the stability and translation of mRNA by directly binding to it or inhibit mRNA expression by recruiting inhibitory complexes such as PRC2 (Polycomb Repressive Complex 2) [26, 27]. For example, lncRNA HOTAIR inhibits the expression of the HOX gene cluster by binding to PRC2 [28], while lncRNA MALAT1 promotes the splicing and stability of specific mRNAs by interacting with splicing factors [29]. The protein SGK1 may play a key role in regulating the proliferation and apoptosis of VSMCs [30]. Studies have shown that SGK1 deficiency attenuated β‐aminopropionitrile‐induced TAD formation and extracellular matrix (ECM) degradation. Mechanistically, SGK1 phosphorylates SIRT6 at Ser338 to induce its ubiquitination‐mediated degradation, thereby relieving SIRT6's transcriptional repression of matrix metalloproteinase 9 (MMP9) through epigenetic modification. This axis critically regulates ECM remodelling and VSMC phenotype switching, with human and murine TAD samples confirming SGK1‐mediated SIRT6‐MMP9 pathway dysregulation. In addition, SGK1 is involved in regulating the cell autophagy process. Mammalian target of rapamycin complex 2 (mTORC2) inhibits the activation of mPTP by binding to SGK1, thereby inducing autophagy. Therefore, SGK1 may be a potential target for AD treatment [31]. Our study found that LINC01605 can promote the expression of SGK1, and they are both located in the cytoplasm, which indicates that LINC01605 may affect the enrichment by regulating mRNA stability or translation. These need further experiments to verify. Moreover, the upregulation of SGK1 can reverse the effects of LINC01605 knockdown on VSMCs. In addition, through Ensembl comparative analysis (https://asia.ensembl.org/Homo_sapiens/Location/Compara_Alignments/Image?align=1960;db=core;g=ENSG00000253161;r=8:37406399‐37625873), we identified four mouse homologues of the human gene LINC01605: ENSMUST00000262045, ENSMUST00000314370, ENSMUST00000210732 and ENSMUST00000297795. The promoter region of human LINC01605 is characterised by dense CpG islands, indicative of strong regulatory potential. Among the mouse homologues, ENSMUST00000297795 exhibits the most similar promoter features to human LINC01605, including shared transcription factor binding sites (e.g., SP/KLF family members such as SP1 and KLF4, as well as CTCF and REST). Furthermore, ENSMUST00000297795 closely resembles human LINC01605 in terms of exon number, length and arrangement. Therefore, in our animal experiments, the gene referred to as LINC01605 corresponds to ENSMUST00000297795. LINC01605 is upregulated in a mouse model of aortic dissection and participates in the cellular processes of migration, proliferation and autophagy associated with aortic dissection by modulating the expression of genes such as SGK1, MMP‐2, MMP‐9, α‐SMA, SM22α, LC3B and p62. Knockdown of LINC01605 can inhibit these processes, suggesting that LINC01605 may be a potential therapeutic target for the treatment of aortic dissection.

Our study also has some limitations. First, the expression of LINC01605 in blood samples was not detected. More evidence is needed for LINC01605 to serve as a clinical biomarker for AAD. Second, the interaction mechanism between LINC01605 and SGK1 needs further research. Whether LINC01605 can act as a sponge lncRNA to affect the expression of other key regulatory factors in AAD has not been fully explored. Nevertheless, our study proposes a new hypothesis for the role of LINC01605 in the progression of AAD.

## 5 Conclusion

In this study, we identified LINC01605 as a novel long noncoding RNA (lncRNA) critically involved in the pathogenesis of aortic dissection (AD). LINC01605 was significantly upregulated in AD tissues and functionally linked to vascular smooth muscle cell (VSMC) dysfunction, including proliferation, migration, phenotypic switching and autophagy dysregulation. Mechanistically, LINC01605 exerts its pathological effects by targeting SGK1, a key regulator of autophagy and VSMC homeostasis. In vitro and in vivo experiments demonstrated that LINC01605 knockdown attenuated Ang II‐induced VSMC dysfunction and aortic injury, while its overexpression exacerbated these effects. These findings highlight the LINC01605‐SGK1 axis as a pivotal regulatory node in AD pathogenesis and underscore the therapeutic potential of targeting lncRNA networks for AD treatment.

## Author Contributions

**Mingliang Li:** conceptualization (lead), data curation (lead), formal analysis (equal), investigation (equal), project administration (equal), writing – original draft (equal), writing – review and editing (equal). **Ruonan Li:** formal analysis (equal), funding acquisition (equal), investigation (equal), methodology (equal). **Zihe Zheng:** investigation (equal), methodology (equal), project administration (equal), software (equal), supervision (equal). **Changbo Xiao:** methodology (equal), software (equal), supervision (equal), validation (equal), visualization (equal). **Quanlin Yang:** investigation (equal), software (equal), supervision (equal), validation (equal), visualization (equal). **Bo Chen:** methodology (equal), software (equal), supervision (equal), validation (equal), visualization (equal). **Xiaofu Dai:** conceptualization (equal), project administration (equal), writing – original draft (equal), writing – review and editing (equal).

## Conflicts of Interest

The authors declare no conflicts of interest.

## Supporting information


**Figure S1:** The impact of LINC01605 on autophagic flux regulation. (A–C) We investigated the protein expression levels of LC3B and p62 under various treatment conditions, including sh‐LINC01605, sh‐LINC01605 + CQ, Ang II + sh‐LINC01605 and Ang II + sh‐LINC01605 + CQ. These measurements were normalised using GAPDH as an internal control. (D, E) Confocal microscopy was employed to visualise the cell nuclei (stained blue with DAPI), LC3‐mRFP (red) and merged images, which provided insights into the formation of autophagosomes under different treatment conditions. (F, G) Further confirmation of autophagosome formation was achieved through confocal microscopy by observing the cell nuclei (DAPI, blue), LC3‐mRFP (red), LC3‐GFP (green) and merged images. This comprehensive analysis elucidated the role of LINC01605 in regulating autophagic flux and its interaction with Ang II‐induced cellular autophagy processes. **p < 0.01 vs. sh‐LINC01605, ^##^ *p* < 0.01 vs. Ang II + sh‐LINC01605.

**Figure S2:** SGK1 promotes proliferation in VSMCs under Ang II‐induced and basal conditions. (A, B) Expression of SGK1 in VSMCs after Ang II treatment (24 h, 1 μM) or transfection with SGK1 siRNA/overexpression plasmid. RT‐qPCR data normalised to GAPDH. (C–H) Proliferation of VSMCs assessed by CCK‐8 assay and EdU. Ang II (1 μM) enhanced proliferation, which was reversed by SGK1 knockdown and further amplified by SGK1 overexpression. Similar trends were observed under basal conditions (no Ang II). **p < 0.01 vs. si‐NC or vector, ^##^ *p* < 0.01 vs. Ang II + si‐NC or Ang II + vector.

**Figure S3:** SGK1 promotes migration in VSMCs under Ang II‐induced and basal conditions. (A–D) The cell migration was detected by transwell migration assays. Ang II stimulation increased VSMC migration, which was exacerbated by SGK1 overexpression and suppressed by knockdown. SGK1 alone (without Ang II) also promoted migration. Representative images (left) and quantification (right). (E–H) The cell migration was detected by scratch assays. **p < 0.01 vs. si‐NC or vector, ^##^ *p* < 0.01 vs. Ang II + si‐NC or Ang II + vector.

**Figure S4:** SGK1 promotes phenotypic transformation and autophagy in VSMCs under Ang II‐induced and basal conditions. (A–P) Western blot analysis of matrix metalloproteinases (MMP‐2, MMP‐9), phenotypic markers (α‐SMA, SM22α) and autophagy‐related proteins (LC3B, p62). SGK1 overexpression downregulated contractile markers (α‐SMA, SM22α) and p62, while upregulating LC3B and MMPs. Knockdown reversed these effects. Quantification normalised to GAPDH. Data shown as mean ± SD. *p < 0.05 vs. si‐NC or vector, **p < 0.01 vs. si‐NC or vector, ^##^ *p* < 0.01 vs. Ang II + si‐NC or Ang II + vector.

**Figure S5:** The regulatory role of LINC01605 in the vascular wall structure of aortic dissection. (A) Elastic van Gieson (EVG) staining was utilised to assess the distribution and integrity of elastic fibres within the vascular wall. (B) Haematoxylin–eosin (HE) staining was employed to examine the cellular composition and inflammatory response within the vascular wall.

**Table S1:** The primer sequences were used for qRT‐PCR.

## Data Availability

The datasets used and/or analysed during the current study are available from the corresponding author on reasonable request.
