# Supplementary figures and images for: LncRNA LINC01605 Regulates Smooth Muscle Cell Functions and Participates in the Development of Aortic Dissection Through Regulating SGK1
Source: J Cell Mol Med. 2025 Nov 26;29(22):e70963. doi: 10.1111/jcmm.70963 (PMC12648298; doi:10.1111/jcmm.70963)

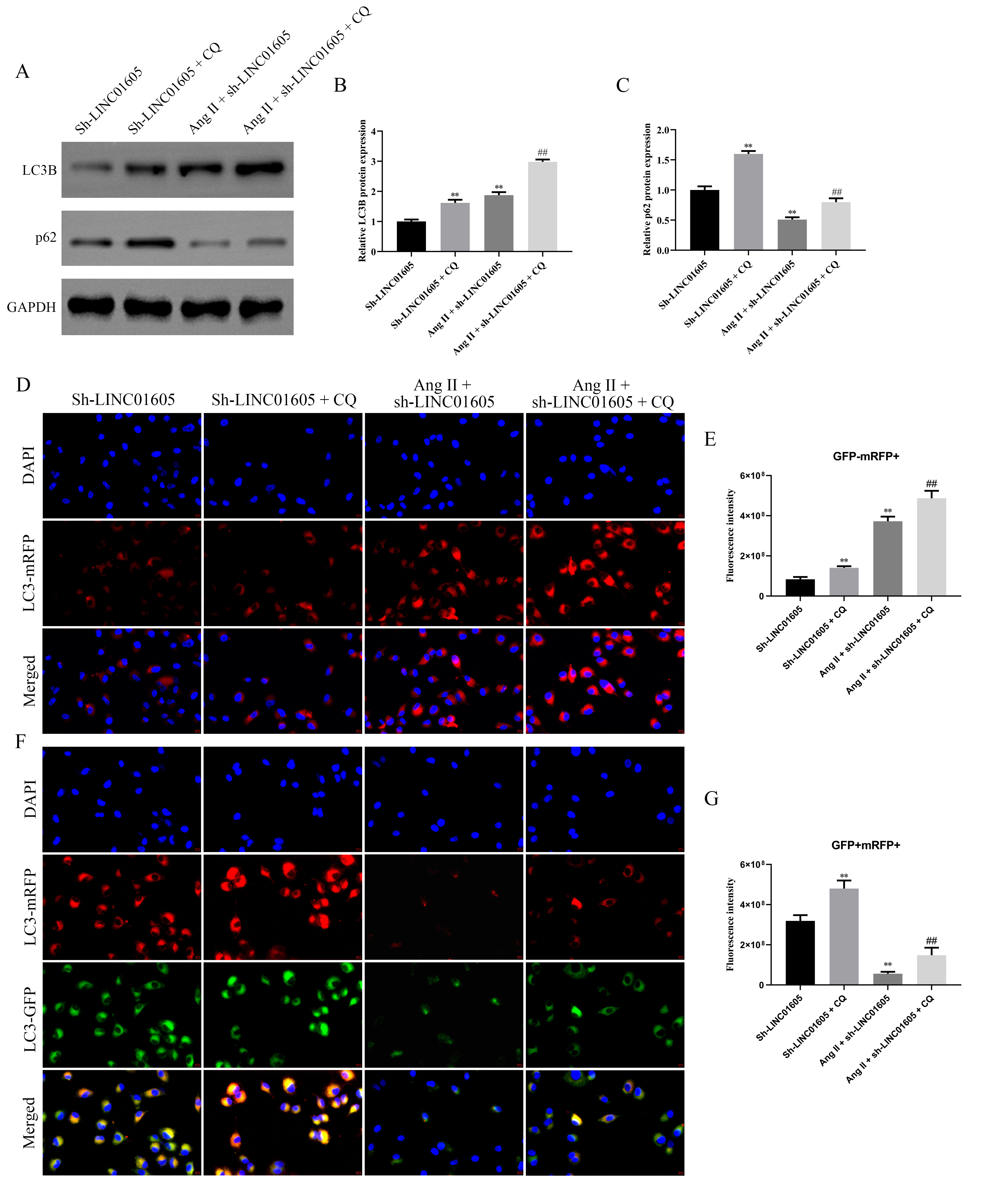

Supplement: Supplementary file 1 — Figure S1: The impact of LINC01605 on autophagic flux regulation. (A–C) We investigated the protein expression levels of LC3B and p62 under various treatment conditions, including sh‐LINC01605, sh‐LINC01605 + CQ, Ang II + sh‐LINC01605 and Ang II + sh‐LINC01605 + CQ. These measurements were normalised using GAPDH as an internal control. (D, E) Confocal microscopy was employed to visualise the cell nuclei (stained blue with DAPI), LC3‐mRFP (red) and merged images, which provided insights into the formation of autophagosomes under different treatment conditions. (F, G) Further confirmation of autophagosome formation was achieved through confocal microscopy by observing the cell nuclei (DAPI, blue), LC3‐mRFP (red), LC3‐GFP (green) and merged images. This comprehensive analysis elucidated the role of LINC01605 in regulating autophagic flux and its interaction with Ang II‐induced cellular autophagy processes. **p < 0.01 vs. sh‐LINC01605, ## p < 0.01 vs. Ang II + sh‐LINC01605. [file JCMM-29-e70963-s003.jpg]

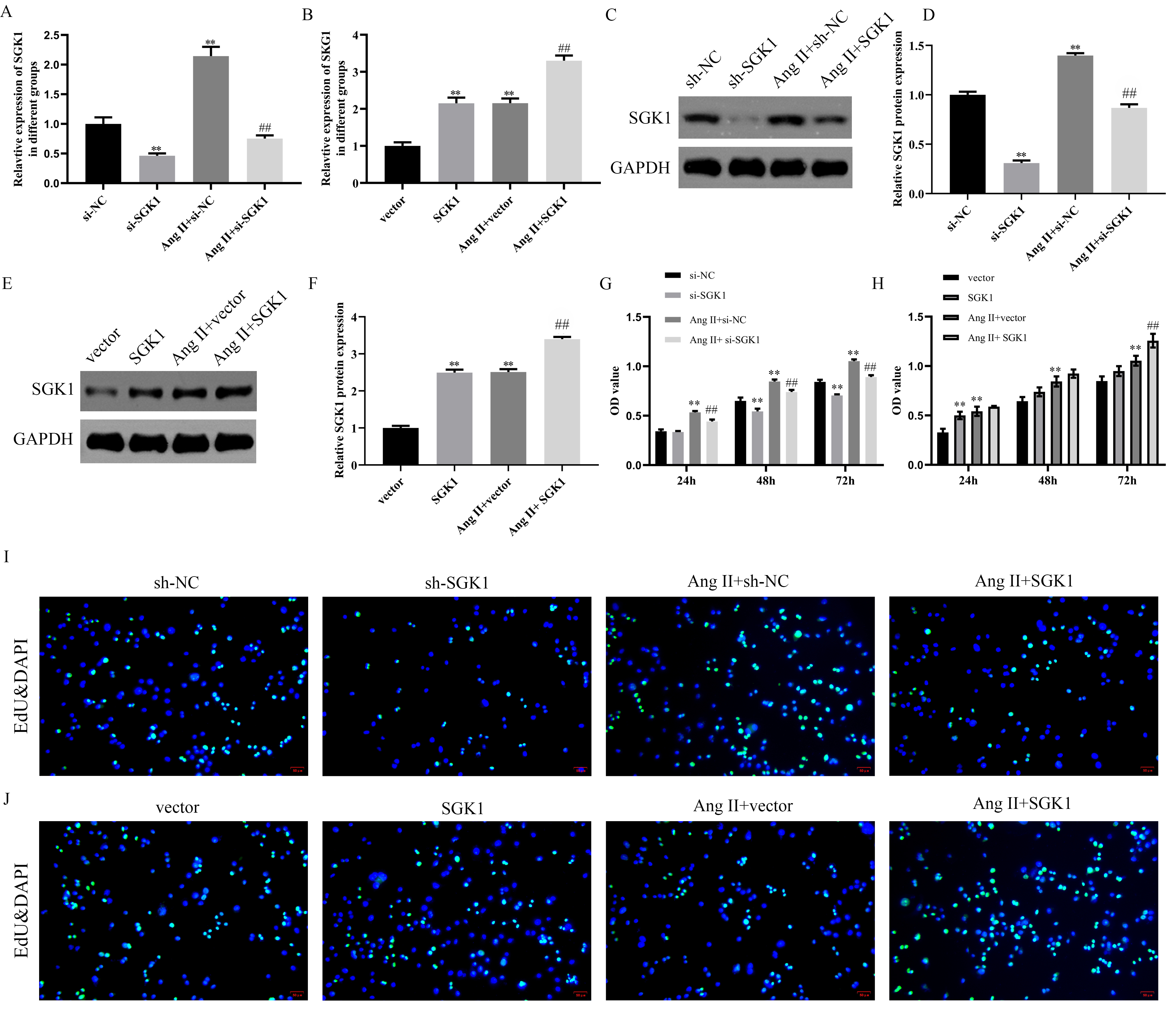

Supplement: Supplementary file 2 — Figure S2: SGK1 promotes proliferation in VSMCs under Ang II‐induced and basal conditions. (A, B) Expression of SGK1 in VSMCs after Ang II treatment (24 h, 1 μM) or transfection with SGK1 siRNA/overexpression plasmid. RT‐qPCR data normalised to GAPDH. (C–H) Proliferation of VSMCs assessed by CCK‐8 assay and EdU. Ang II (1 μM) enhanced proliferation, which was reversed by SGK1 knockdown and further amplified by SGK1 overexpression. Similar trends were observed under basal conditions (no Ang II). **p < 0.01 vs. si‐NC or vector, ## p < 0.01 vs. Ang II + si‐NC or Ang II + vector. [file JCMM-29-e70963-s004.jpg]

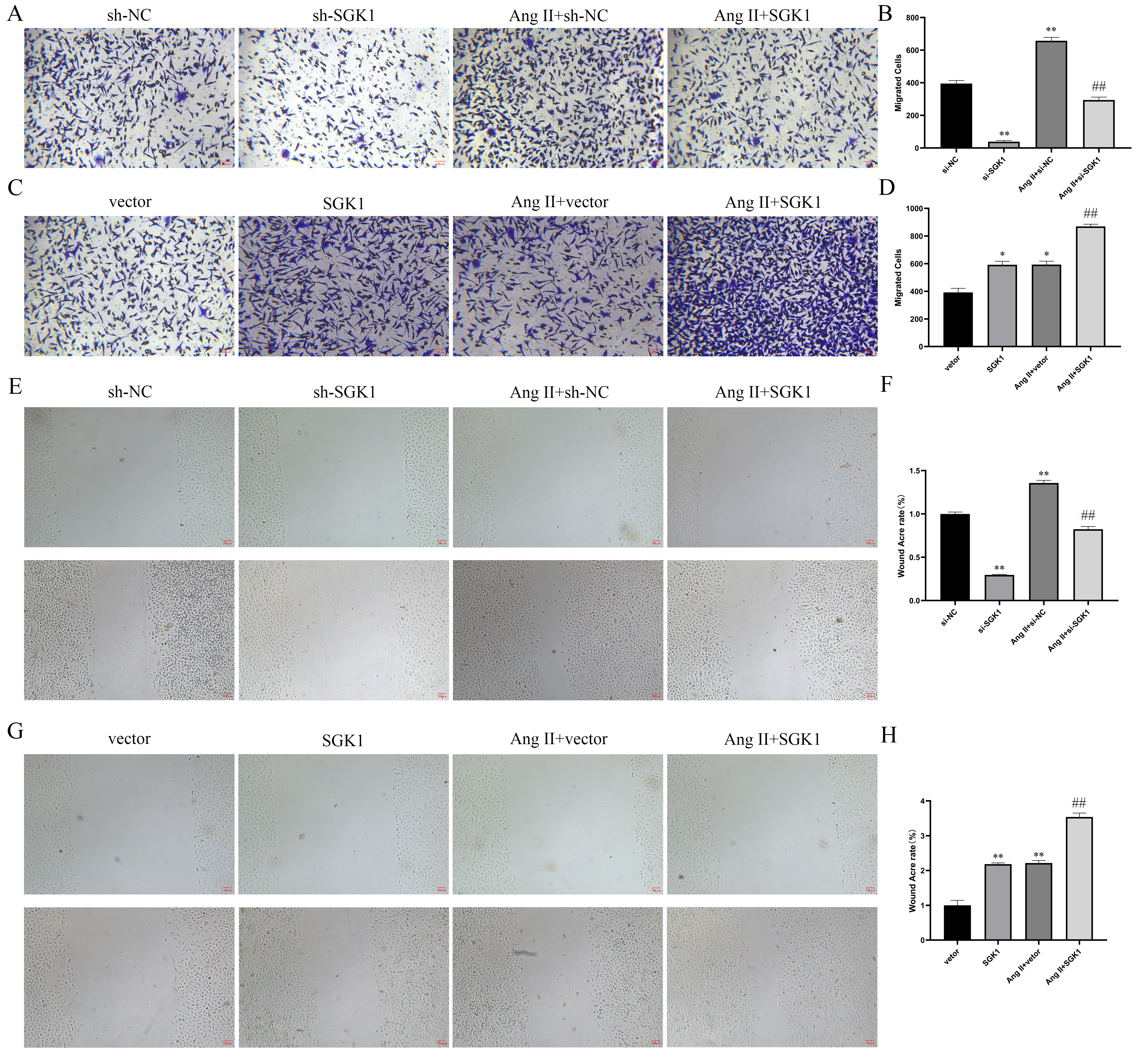

Supplement: Supplementary file 3 — Figure S3: SGK1 promotes migration in VSMCs under Ang II‐induced and basal conditions. (A–D) The cell migration was detected by transwell migration assays. Ang II stimulation increased VSMC migration, which was exacerbated by SGK1 overexpression and suppressed by knockdown. SGK1 alone (without Ang II) also promoted migration. Representative images (left) and quantification (right). (E–H) The cell migration was detected by scratch assays. **p < 0.01 vs. si‐NC or vector, ## p < 0.01 vs. Ang II + si‐NC or Ang II + vector. [file JCMM-29-e70963-s006.jpg]

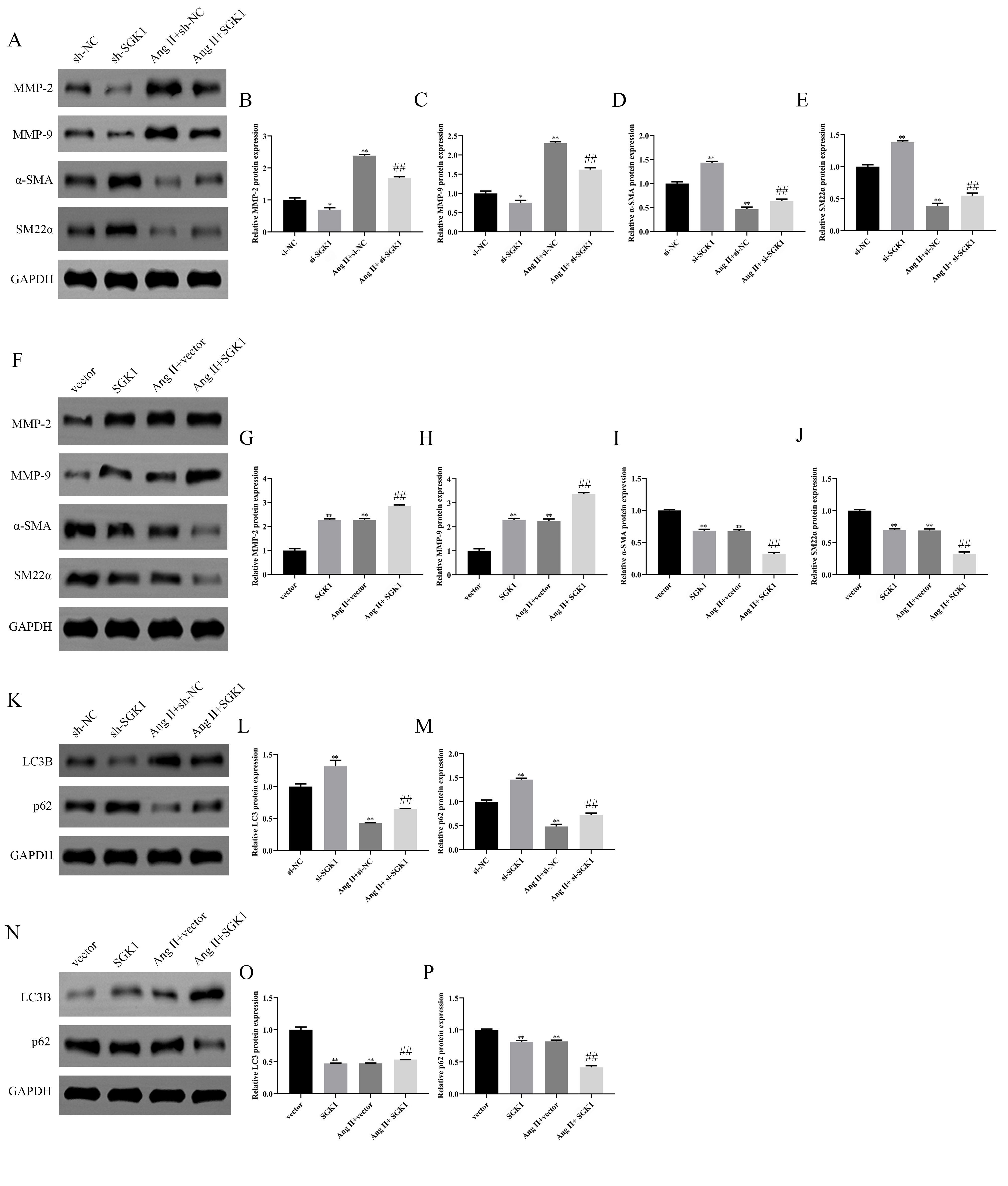

Supplement: Supplementary file 4 — Figure S4: SGK1 promotes phenotypic transformation and autophagy in VSMCs under Ang II‐induced and basal conditions. (A–P) Western blot analysis of matrix metalloproteinases (MMP‐2, MMP‐9), phenotypic markers (α‐SMA, SM22α) and autophagy‐related proteins (LC3B, p62). SGK1 overexpression downregulated contractile markers (α‐SMA, SM22α) and p62, while upregulating LC3B and MMPs. Knockdown reversed these effects. Quantification normalised to GAPDH. Data shown as mean ± SD. *p < 0.05 vs. si‐NC or vector, **p < 0.01 vs. si‐NC or vector, ## p < 0.01 vs. Ang II + si‐NC or Ang II + vector. [file JCMM-29-e70963-s005.jpg]

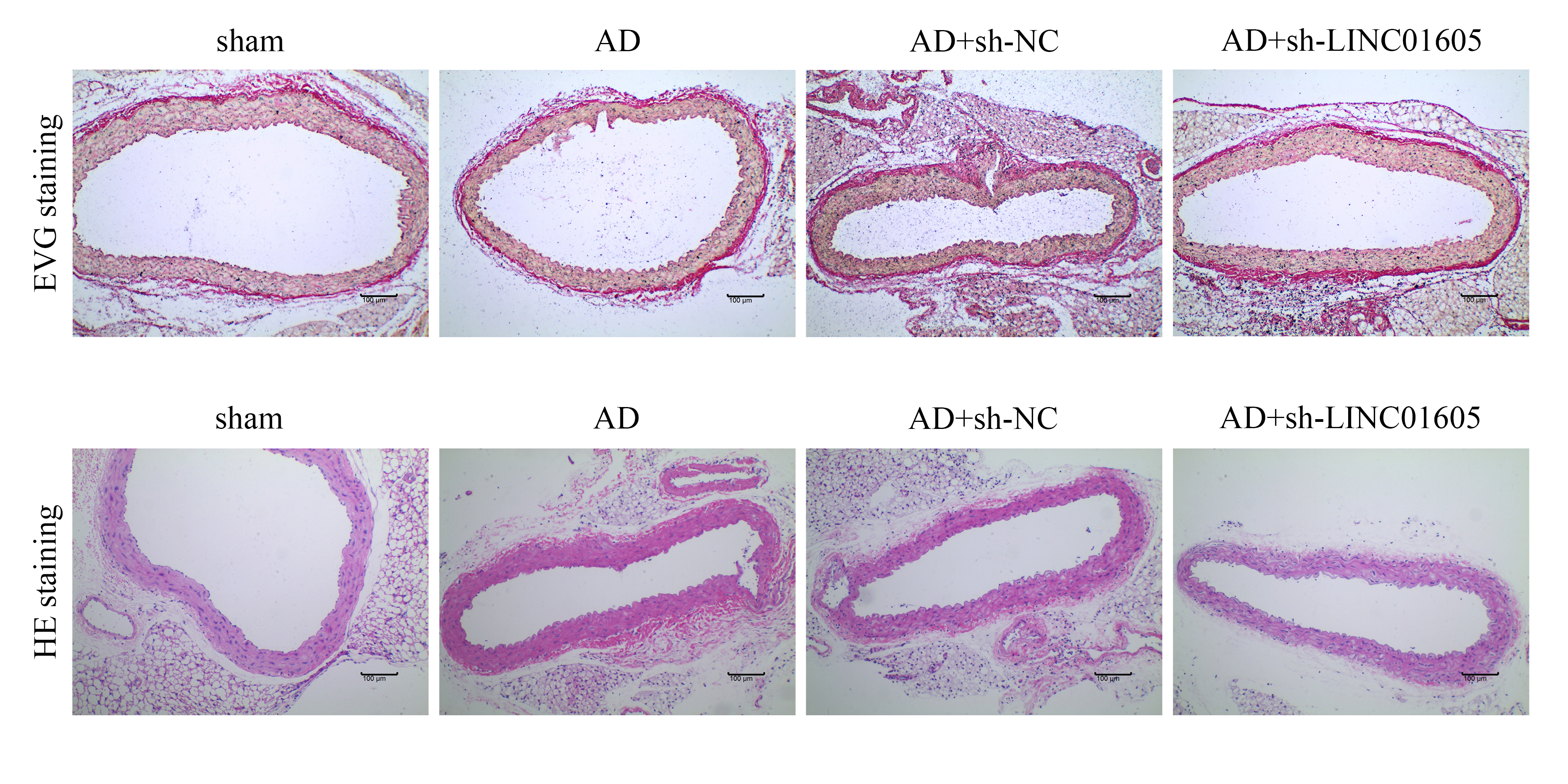

Supplement: Supplementary file 5 — Figure S5: The regulatory role of LINC01605 in the vascular wall structure of aortic dissection. (A) Elastic van Gieson (EVG) staining was utilised to assess the distribution and integrity of elastic fibres within the vascular wall. (B) Haematoxylin–eosin (HE) staining was employed to examine the cellular composition and inflammatory response within the vascular wall. [file JCMM-29-e70963-s002.jpg]
