# Supplementary material for: LncRNA LINC01605 Regulates Smooth Muscle Cell Functions and Participates in the Development of Aortic Dissection Through Regulating SGK1
Source: J Cell Mol Med. 2025 Nov 26;29(22):e70963. doi: 10.1111/jcmm.70963 (PMC12648298; doi:10.1111/jcmm.70963)
Supplement: Supplementary file 6 — Table S1: The primer sequences were used for qRT‐PCR. [file JCMM-29-e70963-s001.docx]

|  | forward | reverse |
| --- | --- | --- |
| β-actin | 5'-GTCCACCGCAAATGCTTCTA-3' | 5'-TGCTGTCACCTTCACCGTTC-3' |
| LINC01605 | 5'-CAACTCATTCCCGTTACAAACA-3' | 5'-CATCTCAACTGCCTCTGTCTCC-3' |
| SGK1 | 5'-GCAGAAGAAGTGTTCTATGCAGT-3' | 5'-CCGCTCCGACATAATATGCTT-3' |
| ENSMUST-00000297795 | 5'-CTGCTTCCTGGTCCTGTTGC-3' | 5'-GAGTTGAATGTGGGTCCTCT-3' |

The following primer sequences were used for qPCR
